# Clinical and Molecular Profiles of a Cohort of Egyptian Patients with Collagen VI-Related Dystrophy

**DOI:** 10.1007/s12031-024-02266-8

**Published:** 2024-10-05

**Authors:** Wessam E. Sharaf-Eldin, Karima Rafat, Mahmoud Y. Issa, Hasnaa M. Elbendary, Noura R. Eissa, Bahaa Hawaary, Nagwa E. A. Gaboon, Reza Maroofian, Joseph G. Gleeson, Mona L. Essawi, Maha S. Zaki

**Affiliations:** 1 Medical Molecular Genetics, Human Genetics and Genome Research Institute, National Research Centre, Cairo, Egypt; 2 Clinical Genetics Department, Human Genetics and Genome Research Institute, National Research Centre, Cairo, 12311 Egypt; 3 Pediatrics Department, Faculty of Medicine, Aswan University, Aswan, Egypt; 4 Medical Genetics Centre, Faculty of Medicine, Ain Shams University, Cairo, Egypt; 5 Medical Genetics Department, Armed Forces College of Medicine, Cairo, Egypt; 6 Department of Neuromuscular Disease, UCL Queen Square Institute of Neurology and the National Hospital for Neurology and Neurosurgery, London, UK; 7 Department of Neurosciences, University of California, San Diego, La Jolla, CA 92093 USA; 8 Rady Children’s Institute for Genomic Medicine, San Diego, La Jolla, CA 92093 USA

**Keywords:** Collagen VI, Bethlem myopathy, Ullrich congenital muscular dystrophy, Next-generation sequencing

## Abstract

Collagen VI-related dystrophies (COL6-RD) display a wide spectrum of disease severity and genetic variability ranging from mild Bethlem myopathy (BM) to severe Ullrich congenital muscular dystrophy (UCMD) and the intermediate severities in between with dual modes of inheritance, dominant and recessive. In the current study, next-generation sequencing demonstrated potential variants in the genes coding for the three alpha chains of collagen VI (*COL6A1*, *COL6A2*, or *COL6A3*) in a cohort of Egyptian patients with progressive muscle weakness (*n* = 23). Based on the age of disease onset and the patient clinical course, subjects were diagnosed as follows: 12 with UCMD, 8 with BM, and 3 with intermediate disease form. Fourteen pathogenic variants, including 5 novel alterations, were reported in the enrolled subjects. They included 3 missense, 3 frameshift, and 6 splicing variants in 4, 3, and 6 families, respectively. In addition, a nonsense variant in a single family and an inframe variant in 3 different families were also detected. Recessive and dominant modes of inheritance were recorded in 9 and 8 families, respectively. According to ACMG guidelines, variants were classified as pathogenic (*n* = 7), likely pathogenic (*n* = 4), or VUS (*n* = 3) with significant pathogenic potential. To our knowledge, the study provided the first report of the clinical and genetic findings of a cohort of Egyptian patients with collagen VI deficiency. Inter- and intra-familial clinical variability was evident among the study cohort.

**Supplementary Information:**

The online version contains supplementary material available at 10.1007/s12031-024-02266-8.

## Introduction

Collagen VI-related dystrophies (COL6-RD) are one of the most common congenital muscle dystrophies (CMDs), accounting for about 30% of cases (Topaloğlu and Poorshiri 2024). They comprise a group of clinically and genetically heterogeneous disorders spanning a clinical continuum from severe Ullrich congenital muscular dystrophy (UCMD; MIM# 254090) to milder Bethlem myopathy (BM; MIM# 158810) with intermediate phenotypes of variable severity (Bozorgmehr et al. 2013). UCMD is characterized by progressive muscle weakness in the neonatal phase or early childhood with prevalent loss of ambulation in the first years of life. However, weakness in BM is expressed by mid-childhood or early adolescence with a low progression rate and retained ambulation into adulthood (Butterfield et al. 2013). Two different phenotypic presentations of BM can be delineated: limb-girdle muscular dystrophy (LGMD), with predomination of muscle weakness, while contractures are either absent or minimal, and myosclerosis with severe contractures without significant weakness (Cruz et al. 2016).

COL6-RD are attributed to pathogenic variants in *COL6A1*, *COL6A2*, and *COL6A3* that code for the three alpha chains, α1, α2, and α3 of Collagen VI, respectively (C. G. Bönnemann 2011). *COL6A1* and *COL6A2* are mapped to 21q22.3, while *COL6A3* is located on 2q37 (Foley et al. 2011). Each alpha chain contains a central triple helical domain (THD) composed of characteristic Gly-X–Y triplets (X and Y are variable amino acids) and flanked by N- and C-terminal globular domains (Kwong et al. 2023). Collagen VI is a protein of the extracellular matrix that virtually exists in all body tissues. In skeletal muscles, it is closely associated with the basement membrane, most probably anchoring the basement membrane into the extracellular matrix (ECM) (Lamandé and Bateman 2018). Assembly of collagen VI starts intracellularly, where monomers (~ 150 nm long) align in an antiparallel manner producing dimers, followed by lateral alignment of dimers to form tetramers. The tetramers are then secreted and arranged extracellularly in an end-to-end pattern creating beaded microfilaments as the end product of the collagen VI assembly (Di Martino et al. 2023).

Both autosomal dominant and autosomal recessive modes of inheritance are associated with Collagen VI deficiency. Dominant negative variants are particularly located in the N-terminal end of the THD disturbing the cysteine residues essential for dimer and tetramer formation resulting in the secretion of non-disulfide bonded tetramers which severely compromise microfibril assembly, whereas the recessive variants are typically nonsense or frameshift variants, or found near the C-terminal of THD or in the C-terminal domain disrupting the initial formation of monomers and preventing their inclusion in the assembly process (Kwong et al. 2023).

BM is usually inherited in an autosomal dominant manner; however, patients with homozygous variants have also been reported. UCMD and intermediate phenotypes are typically autosomal recessive disorders though de novo dominantly acting variants have been characterized in some cases (Picillo et al. 2022). The diagnosis of COL6-RD can be suggested based on the disease’s clinical features. Manifestations of unique muscle changes by magnetic resonance imaging (MRI), and complete or partial loss of collagen VI expression by immunohistochemical studies on muscle biopsy can strongly support the diagnosis. A confirmed analysis can be assessed upon detecting a pathogenic variant in *COL6A1*, *COL6A2*, or *COL6A3* (Bönnemann et al. 2014). This work elucidates the clinical and molecular profiles of a cohort of Egyptian patients with COL6-RD.

## Subjects and Methods

### Clinical Assessment

Twenty-three patients from 17 unrelated Egyptian families were included in this study. They have presented to the Neurogenetics Clinic of the Medical Research Centre of Excellence (MRCE) at the National Research Centre (NRC). The main complaint was weakness of both proximal and distal muscles. All patients were subjected to a complete medical history including three-generation pedigree construction and family and perinatal histories. Basic anthropometric measurements and detailed examination of all body systems were done for all patients. Comprehensive investigations including brain imaging, electroencephalogram (EEG), electromyography (EMG), nerve conduction velocity (NCV), X-rays of spine and hip joint, echocardiogram, and fundus examination were carried out. Serum levels of creatine phosphokinase (CPK) and metabolic workup were also performed.

### Molecular Analysis

Whole exome sequencing (WES) for peripheral blood-derived DNA was carried out using the Illumina platform according to the manufacturer’s protocol. The mean read length was 240 bp with a coverage depth of ˃ 100 × for 95.5% of target bases. Raw fastq reads were aligned to the human reference genome (GRCh37/hg19) using the BWA-MEM algorithm. The aligned reads were saved in SAM format, and then converted to BAM format using Picard. The Genome Analysis ToolKit (GATK) was utilized to generate the VCF files containing the different genetic variants that were annotated based on their gene context using Ensembl’s Variant Effect Predictor (VEP). The pathogenicity of the identified variants was assessed based on the American College of Medical Genetics (ACMG) guidelines (Richards et al. 2015). Variants were checked in reference population databases, where novel variants were submitted to ClinVar. For familial segregation, specific primer pairs were designed using the available genomic sequences, and target sequences were analyzed.

## Results

### Clinical Results

**Fig. 1:**
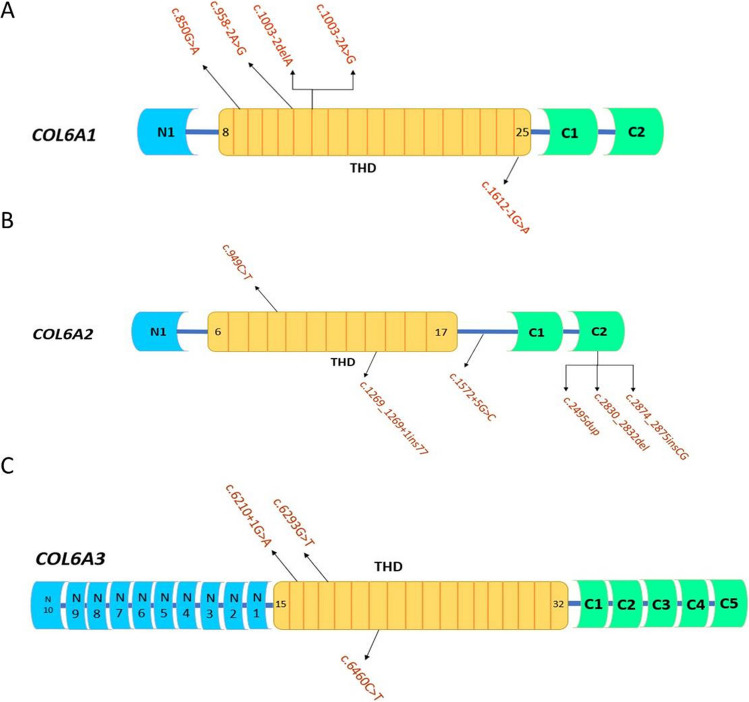
Schematic representation of variants reported in the study cohort. Monoallelic variants are indicated above the scheme, while biallelic variants are indicated below. THD, triple helical domain; C, C-terminus; N, N-terminus

The clinical features of the enrolled subjects were summarized in Table 1 and detailed in supplementary Table 1. The study involved 14 males and 9 females with ages ranging from 1 to 15 years old. Parental consanguinity and family history were positive in 13 (76.5%) and 9 (52.9%) families, respectively (Fig. 1 Supplementary). Most of our patients originated from the governorates of Upper Egypt (64.7%). Anthropometric measurements were normal in most subjects. However, short stature and microcephaly were reported in only one patient per each (P7 and P8, respectively) and 5 patients were considered underweight (21.7%).

**Table 1:** Clinical findings of the enrolled subjects

Feature	Prevalence
Gender, no of males (%)–no of females (%)	14 (60.9)–9 (39.1)
Consanguinity, no of families (%)	13 (76.5)
Family history, no of families (%)	9 (52.9)
Origin, no of families (%)	
Cairo	3 (17.6)
Lower Egypt	3 (17.6)
Upper Egypt	11 (64.7)
Diagnosis, no of patients (%)	
BM	8 (34.8)
UCMD	12 (52.2)
Intermediate	3 (13)
Fetal movement, no of patients (%)	
Normal	12 (52.2)
Weak	11 (47.8)
Anthropometry, mean SD (minimum—maximun)	
Weight	− 1.8 (− 4.4–0.9)
Height	− 1.1 (− 3.2–1.1)
Head circumference	− 1.1 (− 3.1–1.5)
Onset of symptoms (months) mean ± SD (minimum–maximum)	12.2 ± 14 (0–60)
Delayed motor development, no of patients (%)	13 (56.5)
Motor regression, no of patients (%)	4 (17.4)
Ambulation, no of patients (%)	13 (56.5)
Defective cognition, no of patients (%)	1 (4.3)
Hypotonic facies, no of patients (%)	11 (47.8)
Hypotonia, no of patients (%)	23 (100)
Hyporeflexia, no of patients (%)	23 (100)
Proximal muscle weakness, no of patients (%)	23 (100)
Distal muscle weakness, no of patients (%)	23 (100)
Neck torticollis, no of patients (%)	4 (17.4)
Skeletal deformities, no of patients (%)	17 (73.9)
Hyperextensibility, no of patients (%)	23 (100)
Respiratory involvement, no of patients (%)	4 (17.4)
Long fingers and toes, no of patients (%)	11(47.8)
Talipes, no of patients (%)	2 (8.7)
Abnormal neuroimaging, no of patients (%)	2 (8.7)
Myopathic EMG/NCV, no of patients (%)	23 (100)
CPK mean ± SD (minimum–maximum)	387.9 ± 533.5 (68–2791)

Muscle weakness could be indicated during pregnancy, as reduced fetal movements in nearly half of the patients (11/23: 47.8%). Delayed motor milestones were noticed in 13 patients (56.5%), and 6 patients were never achieved walking independently (26.1%). Motor skills were regressed in 4 patients (17.4%) between the ages of 3 to 5 years old and they lost their ability to walk. Thirteen patients (56.5%) were still ambulant with a waddling gait throughout their last examination. Proximal and distal muscle weakness was evident in all subjects with the predominance of proximal muscle affection. Accordingly, neurological examination identified axial hypotonia and hyporeflexia in all patients. Neck torticollis was assessed in 4 cases (17.4%). Hyperextensibility, the most pathognomonic finding of the disease was reported in all patients. Myopathic changes were detected in all patients utilizing EMG and NCV. Mildly elevated CPK levels ( < 500 units/L) were reported in 20 subjects (87%). None of our patients was subjected to muscle biopsy.

Normal cognitive function was observed in the majority of cases (95.6%) except for only one patient (P6) with mild defective speech and learning disability (IQ:73). Some facies were common among the enrolled subjects including hypotonic facies (11 patients; 47.8%), and large prominent ears and/or low set ears (4 patients; 17.3%). Other less common facies were sparse hair, upturned nose, pointed chin, and preauricular ear tags. Skeletal abnormalities were also prominent among patients, including rocker bottom feet (14 patients; 60.8%), severe acquired arthrogryposis (9 patients; 39.1%), and scoliosis (4 patients; 17.3%). X-rays of the spine and hip joints showed scoliosis, kyphosis, and lumbar lordosis in the examined subjects. Other systemic manifestations included tachycardia, tricuspid regurgitation, or atrial septal defect (ASD) in 5 patients (21.7%) and recurrent chest infection in 4 patients (17.3%). Neuroimaging was normal in all patients except for two who had mild patchy white matter signal (P10), and thin corpus callosum with mild vermian hypoplasia (P23).

In our case series, 3 families (F1, F4, and F10) showed significant interfamilial variability. Family 1 with two affected brothers (P1, P2) diagnosed with UCMD and intermediate phenotype, respectively. Patient 1 lost the ability to walk at the age of 3 years and developed scoliosis and knee arthrogryposis while P2 is still ambulant with a waddling gait and limping. The 2 affected relatives of family 4 (P5 and P6) presented with remarkable variable expressivity. Patient 5 presented with floppiness at the age of 1 month and was never achieved standing or walking and acquired early knee arthrogryposis whereas patient 6 developed the first disease symptoms at the age of 7 months and was still ambulant with mild flexed knees. Similarly, family 10 (P13, 14) with UCMD had considerable clinical variability. Patient 13 exhibited delayed walking with onset at 3 years and stationary course, while the brother (P14) had normal motor development with notable regression at the age of 5 years after COVID-19 infection and lost the ability to walk. Other families with 2 affected sibs (F5, F11, and F12) showed irrelevant clinical heterogeneity.

### Molecular Results

A total of 14 disease-causing variants have been identified in the enrolled families (*n* = 17) (Table 2, Fig. 1 Supplementary). They included five variants in *COL6A1*, six variants in *COL6A2*, and three variants in *COL6A3* (Fig. 1). Seven homozygous variants, including one nonsense, one inframe, two splicing, and three frameshift, were identified in 13 patients with the recessive disease form. The splicing variants, c.1612-1G > A and c.1572 + 5G > C, result in the skipping of exons 25 of α1 and 19 of α2, respectively. These patients were mainly diagnosed as UCMD (*n* = 8) and less commonly as intermediate phenotype (*n* = 3) or BM (*n* = 2). They were either found in the C-terminal end of THD, the C-terminus, or led to early protein truncation in THD. They were mainly mapped to *COL6A2* (*n* = 5). Four homozygous variants were novel, classified as likely pathogenic (*n* = 3) or VUS (*n* = 1), where the latter (NM_001849.4:c.1572 + 5G > C) was a donor splice site that was predicted to have a damaging effect on protein function (SpliceAl = 0.96). Another VUS variant, p.(Phe944del), was an inframe deletion in the C-terminus of *COL6A2*. The variant allele frequency on gnomAD (v4.1.0) is 0.000002489; however, it has not been reported as homozygous in the database. ClinVar database had a previous entry for this variant with uncertain effect. The variant was predicted to be deleterious by both MutationTaster (Schwarz et al. 2014) and PROVEAN (score =  − 5.05) (Choi and Chan 2015). Further, it was well segregated in the available family members, where patients’ parents were heterozygous and other healthy siblings were either carrier or variant-free.

**Table 2:** Molecular results of the enrolled subjects

Family (patient)	Gene	Genomic position (GRCh38)	Nucleotide change	Protein change	Exon/Intron	Domain	Variant type	ACMG classification (criteria)	Zygosity	Allele frequency (gnomAD v4.1.0)	REVEL	SpliceAl	Diagnosis
F1 (P1, P2)	*COL6A2*	chr21-46,131,986 G > GT	c.2495dup*	p.(Asp833Glyfs*14)	**Exon** 28	C-terminus	Frameshift	Likely pathogenic (PS4, PVS1, PM2)	Homozygous	Not found	NA	NA	P1: UCMD P2: Intermediate
F2 (P3)	*COL6A2*	chr21-46,132,320 CCTT > C	c.2830_2832del	p.(Phe944del)	**Exon** 28	C-terminus	Inframe deletion	VUS (PM2, PM4)	Homozygous	0.000002489	NA	NA	P3: UCMD
F3 (P4)	*COL6A2*	chr21-46,119,119 G > GCATCATCCCTCTACATTTTTTAACCAAATCAACAACAACCTATTTAGCTGTTCCCCAACCTTTTCCTCCGACAGCCG	c.1269_1269 + 1ins77*	p.(Gly424Hisfs*147)	**Exon** 14	THD	Frameshift	Likely pathogenic (PVS1, PM2)	Homozygous	Not found	NA	NA	P4: Intermediate
F4 (P5, P6)	*COL6A2*	chr21-46,132,320 CCTT > C	c.2830_2832del	p.(Phe944del)	**Exon** 28	C-terminus	Inframe	VUS (PM2, PM4)	Homozygous	0.000002489	NA	NA	P5: UCMD P6: Intermediate
F5 (P7, P8)	*COL6A2*	chr21-46,132,320 CCTT > C	c.2830_2832del	p.(Phe944del)	**Exon** 28	C-terminus	Inframe	VUS (PM2, PM4)	Homozygous	0.000002489	NA	NA	P7: UCMD P8: UCMD
F6 (P9)	*COL6A3*	chr2-237,358,532 G > A	c.6460C > T	p.(Arg2154*)	**Exon** 21	THD	Nonsense	Pathogenic (PVS1, PM2, PP5)	Homozygous	0.000007953	NA	NA	P9: BM
F7 (P10)	*COL6A1*	chr21-45,989,129 G > A	c.850G > A	p.(Gly284Arg)	**Exon** 9	THD	Missense	Pathogenic (PS2, PM2, PP3, PM1)	Heterozygous (de novo)	0.000	0.98 (strong pathogenic)	NA	P10: UCMD
F8 (P11)	*COL6A3*	chr2-237,361,120 C > T	c.6210 + 1G > A	p.(?)	**Intron** 16	THD	Splice donor	Pathogenic (PVS1, PS2, PM2)	Heterozygous (de novo)	Not found	NA	1 (strong)	P11: UCMD
F9 (P12)	*COL6A2*	chr21-46,116,672 C > T	c.949C > T*	p.(Arg317Cys)	**Exon** 9	THD	Missense	VUS (PM2)	Heterozygous (de novo)	0.000009299	0.6 (uncertain)	NA	P12: UCMD
F10 (P13, P14)	*COL6A2*	chr21-46,132,366 C > CCG	c.2874_2875insCG*	p.(Glu959Argfs*82)	**Exon** 28	C-terminus	Frameshift	Likely pathogenic (PS4, PVS1, PM2)	Homozygous	Not found	NA	NA	P13: UCMD P14: UCMD
F11 (P15, P16)	*COL6A1*	chr21-45,990,770 CA > C	c.1003-2delA	p.(?)	**Intron 13**	THD	Splice acceptor	Pathogenic (PS3, PSV1, PM2)	Heterozygous (paternally inherited)	Not found	NA	0.98 (strong)	P15: BM P16: BM
F12 (P17, P18	*COL6A1*	chr21-45,990,771 A > G	c.1003-2A > G	p.(?)	**Intron 13**	THD	Splice acceptor	Pathogenic (PSV1, PM2)	Heterozygous (maternally inherited)	Not found	NA	0.98 (strong)	P17: BM P18: BM
F13 (P19)	*COL6A3*	chr2-237,359,378 C > A	c.6293G > T	p.(Gly2098Val)	**Exon** 18	THD	Missense	Likely pathogenic (PM2, PM5, PP3)	Heterozygous (de novo)	Not found	0.9 (moderate pathogenic)	NA	P19: BM
F14 (P20)	*COL6A1*	chr21-45,998,896 G > A	c.1612-1G > A	p.(?)	**Intron 24**	THD	Splice acceptor	Pathogenic (PSV1, PM2, PP5)	Homozygous	Not found	NA	0.99 (strong)	P20: BM
F15 (P21)	*COL6A1*	chr21-45,990,376 A > G	c.958-2A > G	p.(?)	**Intron 12**	THD	Splice acceptor	Pathogenic (PSV1, PS2, PM2)	Heterozygous (de novo)	Not found	NA	0.99 (strong)	P21: UCMD
F16 (P22)	*COL6A2*	chr21-46,122,163 G > C	c.1572 + 5G > C*	p.(?)	**Intron 19**	C-terminus	Splice donor	VUS (PM2, PP3)	Homozygous	Not found	NA	0.96 (strong)	P22: UCMD
F17 (P23)	*COL6A1*	chr21-45,989,129 G > A	c.850G > A	p.(Gly284Arg)	**Exon** 9	THD	Missense	Pathogenic (PS2, PM2, PP3, PM1)	Heterozygous (de novo)	0.000	0.98 (strong pathogenic)	NA	P23: BM

The asterisk (*) denotes for novel variant. The reference sequences of *COL6A1*, *COL6A2*, and *COL6A3* are NM_001848.3, NM_001849.4, and NM_004369.4, respectively. REVEL is an ensemble method for predicting the pathogenicity of missense variants based on a combination of scores from 13 individual tools: MutPred, FATHMM v2.3, VEST 3.0, PolyPhen-2, SIFT, PROVEAN, MutationAssessor, MutationTaster, LRT, GERP +  + , SiPhy, phyloP, and phastCons. REVEL was trained using recently discovered pathogenic and rare neutral missense variants, excluding those previously used to train its constituent tools. The REVEL score for an individual missense variant can range from 0 to 1, with higher scores reflecting a greater likelihood that the variant is disease-causing. Based on ClinGen’s recommendation, score above 0.932 corresponds with strong pathogenic evidence, score in the range (0.773, 0.932) moderate pathogenic, score in the range (0.644, 0.773) supporting pathogenic, score in the range (0.183, 0.29) supporting benign, score in the range (0.016, 0.183) moderate benign, score in the range (0.003, 0.016) strong benign, and score below 0.003 Very strong benign. SpliceAI uses deep neural networks to predict whether splicing events occur. The score can range from 0 to 1, when scores can be interpreted as the probability of the variant being splice-altering

Ten patients (6 BM and 4 UCMD) had heterozygous dominantly acting variants in the N-terminus of THD, including 3 missense and 4 splicing variations. Two missense variants in 3 patients were located in the Gly-X–Y motif. The 2 splice variants in family 11 and family 12 were paternally and maternally transmitted, respectively. Unlike the gene distribution of the recessive variants, four heterozygous variants are located in *COL6A1*, two in *COL6A3*, and only one in *COL6A2*. They have no allele frequency in the reference population databases and are classified as pathogenic or likely pathogenic except for p.Arg317Cys in *COL6A2* which has uncertain significance. The variant MetaRNN score is 0.82 (Li et al. 2022). MetaRNN score ranges from 0.0 to 1.0, where a higher score is more likely to be pathogenic. Wild-type and mutant amino acids are different in size, charge, and hydrophobicity, possibly disturbing correct protein folding and/or affecting interactions with other molecules (Venselaar et al. 2010).

All detected variants were reported in only one family, except for *COL6A1* p.(Gly284Arg) and *COL6A2* p.(Phe944del); they were identified in 2 and 3 families, respectively. All patients (*n* = 5) with p.(Phe944del) manifested a relatively severe phenotype (4 UCMD and 1 intermediate form). However, p.(Gly284Arg) was identified in a heterozygous state in 2 subjects (P10 and P23) from different families with significant clinical heterogeneity. P10 exhibited delayed motor development presenting UCMD. He walked at 4.5 years with flexed knees, acquired arthrogryposis, and rocker bottom feet. On the contrary, patient 23 was diagnosed as BM with normal motor milestones and clinically manifested with mild weakness and hyperextensibility.

## Discussion

Collagen VI protein anchors muscles, cartilage, and skin to the ECM. Therefore, pathogenic mutations in genes providing the blueprints for collagen VI cause instability of the ECM in these tissues. In turn, they impact their structure and function resulting in varying degrees of muscle weakness, joint contractures, and other symptoms (Williams et al. 2022). In the current study, we elucidated the clinical and genetic profile of a cohort of Egyptian patients with COL6-RD. As far as we know, this is the first demonstration of collagen VI deficiency in Egypt and other Arab countries. The study included 23 patients from 17 unrelated families, where WES was employed for patients’ molecular analysis. The genetic heterogeneity of collagen VI-related dystrophy and variant distributions all over the involved genes strengthen the application of high through-output techniques in the disease molecular diagnosis (Marinella et al. 2022).

There is a scarcity of the prevalence of COL6-RD disorders. In a study from northern England, the prevalence of BM and UCMD was calculated as 0.77 and 0.13 per 100,000, respectively (Norwood et al. 2009). In Japan, a higher incidence of UCMD was estimated at 0.20 in 100,000 births (Inoue et al. 2021). The frequency of BM and UCMD was variable among different studies with relatively few cases of the intermediate phenotype described by early onset ( < 2 years) but slowly progressive disease course lacking the typical characteristics of both BM and UCMD (Allamand et al. 2010). In the study cohort, UCMD was more prevalent. The enrolled subjects were diagnosed as 12 UCMD (52.2%), 8 BM (34.8%), and 3 with intermediate presentation (13%).

The enrolled subjects presented with the hallmarks of COL6-RD including muscle weakness, proximal contractures, and joint hyperlaxity. Patients with BM (*n* = 8) achieve ambulation with mild or absent motor developmental delay. Age of onset ranged from 6 months to 5 years with slow disease progression. In patients with UCMD, disease manifestations were noticeable during the first year of life. Seven children were never able to walk independently, and ambulation had already been lost in the other 3 patients at their last examination. Similarly, the onset of the intermediate form occurred in early infancy ranging from 3 to 12 months, but with less motor and respiratory impairment compared to UCMD.

More than 2000 different pathogenic variants have been reported in the genes encoding the three subunits of collagen IV, where *COL6A2* and *COL6A3* variants are more predominant. Based on the Human Gene Mutation Database (HGMD), point mutations in the three genes, including missense, nonsense, and splicing mutations, account for ≈82% of variants. Other variations include small frameshift mutations (≈13%) and less frequently large genomic rearrangements (≈5%) (Stenson et al. 2014). In the current study, the reported variants (*n* = 14) were scattered as 5 in *COL6A1*, 6 in *COL6A2*, and 3 in *COL6A3* in 6, 8, and 3 families, respectively. They comprised 3 missense, 3 frameshift, and 6 splicing variations as well as an inframe deletion and a nonsense alteration, including 5 variations that were not previously reported. Three VUS variants (1 missense, 1 splice-site, and 1 inframe) were reported in 7 patients compatible with COL6-RD. No other clinically relevant variants were identified in these cases. The current VUS variants were predicted to affect the protein structure and/or function using comparable online tools and segregated properly in the available family members.

The limitation of the present study is the inability to perform 2 mandatory investigations, muscle neuroimaging, and muscle biopsy on our patients. Both could greatly help to differentiate certain neuromuscular diseases specifically COL6-RD that showed particular patterns of muscle involvement. However, these procedures are not carried out as routine investigations in Egypt due to their high expenses and the scarcity of specialized centers. Further, in our community, a potentially invasive procedure like muscle biopsy is often met with rejection by patients’ guardians.

Among the enrolled subjects, the number of patients with recessive variants (56.5%) is slightly higher than that with dominant variants (43.5%). Notably, in the largest cohort of collagen VI deficiency, dominant cases were more prevalent. Inoue et al. described 147 Japanese patients, where only 11 cases (7.5) revealed a recessive mode of inheritance (Inoue et al. 2021). In a multicenter study from Spain and USA, a total of 84 patients (70.6%) carried mono-allelic variants, while 31 patients (26.1%) carried bi-allelic variations (Natera-de Benito et al. 2021). A study of ≈50 patients from different origins also reported a higher number of patients with heterozygous variants (> 60%) (Briñas et al. 2010). Even in the smaller studies, patients carrying dominantly acting variants seem more prevalent. However, in a study of 25 patients from a single French center, the numbers of patients with dominant and recessive were quite similar (Morel et al. 2023), consistent with our results. The slight predominance of the recessive presentation could be attributed to the higher rate of consanguinity in our community, especially in upper Egypt (> 40%) (Ahmed 2017).

Genetic defects associated with BM are mainly dominant and the most common causative variants are splice site alterations causing skipping of exon 14 of *COL6A1* and missense variants affecting glycine residues in the Gly-X–Y motif (Lee et al. 2017). In the current study, we identified de novo missense and splice site variants in 4 (F7, F9, F13, and F17) and 2 families (F8, and F15), respectively. Another 2 splice site variants were inherited from one of their parents (F11, and F12). Six out of the 8 cases diagnosed to have BM had dominantly acting variants (75%), where skipping of *COL6A1* exon 14 and substitution of conserved glycine residues were reported in 4 and 2 patients, respectively. UCMD and intermediate phenotypes are commonly inherited in an autosomal recessive manner (Picillo et al. 2022). Among patients diagnosed as intermediate or UCMD (*n* = 15), 12 subjects harbored bi-allelic recessive variants (80%) including 3 frameshift variants as well as 2 splice-site alterations and 1 inframe deletion. Noteworthy, homozygous variants causing premature protein truncation are known as a major cause of more severe phenotypes (Natera-de Benito et al. 2021).

The crucial cysteine residues for disulfide bonding during dimer and tetramer assembly are located in exon 14 of α1, exon 11 of α2, and exon 17 of α3. Variants causing the deletion of these cysteine residues prevent the mutant chain from subsequent participation in the assembly process, resulting in limited dominant negative effect and a mild phenotype (Kwong et al. 2023). In the study cohort, 2 variants in *COL6A1* (c.1003-2delA and c.1003-2A > G) resulted in the skipping of exon 14 in 4 patients with BM fitting into the associated phenotype-genotypes correlations. Conversely, skipping variants preceding the crucial cysteine residues in THD allow further incorporation of the mutant chains into dimers and tetramers, causing a strong dominant negative effect on the collagen VI matrix and severe phenotype (Lampe et al. 2008). Consistently, the variants c.958-2A > G and c.6210 + 1G > A were reported in 2 patients with UCMD among the enrolled subjects causing skipping of exon 13 of α1 and exon 16 of α3, respectively.

Inter- and intrafamilial as well as intergenerational phenotypic variability and disease progression are also usual in the COL6-RD (Bardakov et al. 2021). This might be attributed to unknown genetic modifiers and/or environmental factors. For example, the *COL6A1* p.Gly284Arg variant was previously identified in a heterozygous state in patients encompassing a range of disease severity from mild BM and early onset severe UCMD (Yonekawa and Nishino 2014). This variant was also reported twice among the study cohort with extreme clinical variability.

## Conclusion

Identifying a pathogenic variant in a gene, coding for a collagen VI subunit, in patients with neuromuscular disorders is the gold standard method for COL6-RD diagnosis. The present study provided insights into the clinical and the molecular spectrum of COL6-RD in Arab populations strengthening the application of comprehensive strategies for the molecular analysis of these group disorders. Inter- and intra-familial clinical variability has been distinguishable between some patients.

## Supplementary Information

Below is the link to the electronic supplementary material.

Supplementary file1 Figure 1 Supplementary: Family pedigree of the 23 studied patients with collagen VI related dystrophy. New variants are identified by the symbol #. Segregation of the variant is shown on the pedigree as +/+, +/-, and -/- (with + for normal and - for mutated). The autosomal dominant pattern of the disorder is present in 8 families (F7, F8, F9, F11, F12, F13, F15, and F17) with de novo variants in 6 families (F7, F8, F9, F13, F15, and F17) and parentally inherited in 2 families (F11, and F12). Autosomal recessive inheritance is present in 9 families (F1-F6, F10, F14, and F16). (JPG 346 KB)

Supplementary file2 (XLSX 26 KB)

## Data Availability

No datasets were generated or analysed during the current study.
