# Supplementary figures and images for: Clinical and Molecular Profiles of a Cohort of Egyptian Patients with Collagen VI-Related Dystrophy
Source: J Mol Neurosci. 2024 Oct 5;74(4):93. doi: 10.1007/s12031-024-02266-8 (PMC11452470; doi:10.1007/s12031-024-02266-8)

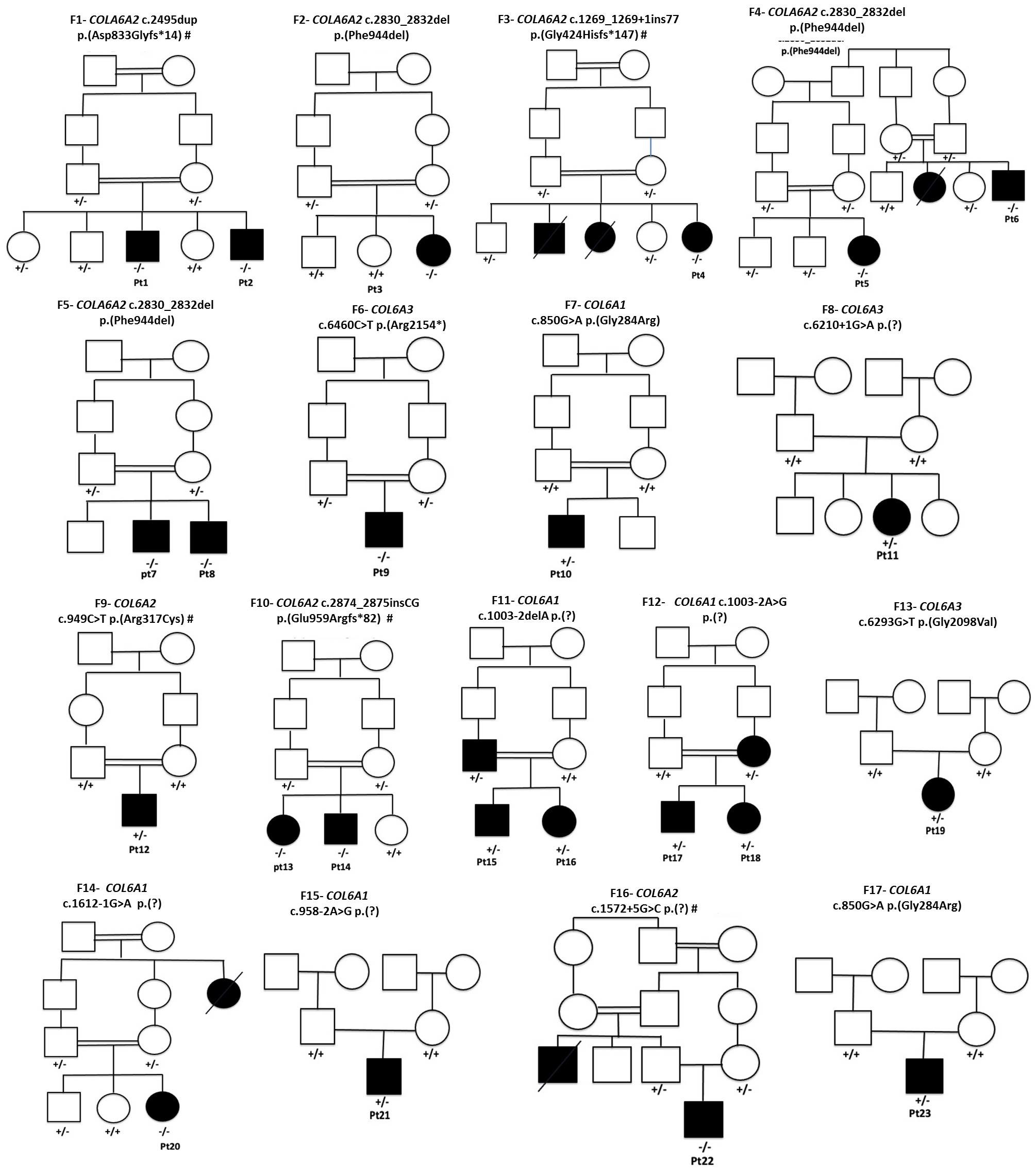

Supplement: Supplementary file 1 — Supplementary file1 Figure 1 Supplementary: Family pedigree of the 23 studied patients with collagen VI related dystrophy. New variants are identified by the symbol #. Segregation of the variant is shown on the pedigree as +/+, +/-, and -/- (with + for normal and - for mutated). The autosomal dominant pattern of the disorder is present in 8 families (F7, F8, F9, F11, F12, F13, F15, and F17) with de novo variants in 6 families (F7, F8, F9, F13, F15, and F17) and parentally inherited in 2 families (F11, and F12). Autosomal recessive inheritance is present in 9 families (F1-F6, F10, F14, and F16). (JPG 346 KB) [file 12031_2024_2266_MOESM1_ESM.jpg]
